# Editorial: Interdisciplinary synergies in neuroinformatics, cognitive computing, and computational neuroscience

**DOI:** 10.3389/fncom.2025.1657167

**Published:** 2025-08-06

**Authors:** Nabamita Deb, Zardad Khan, Muhammad Sulaiman, Maharani Abu Bakar

**Affiliations:** ^1^Department of Information Technology, Gauhati University, Guwahati, India; ^2^Department of Statistics and Business Analytics, United Arab Emirates University, Al-Ain, United Arab Emirates; ^3^Department of Mathematics, Abdul Wali Khan University Mardan, Mardan, Pakistan; ^4^Department of Applied Mathematics, Universiti Malaysia Terengganu, Kuala Terengganu, Malaysia

**Keywords:** synergies in neuroinformatics, cognitive computing, computational neuroscience, affective computing, brain computer interface

“Converging Minds: Synergies in Neuroinformatics, Cognitive Computing, and Computational Neuroscience”

The past years have seen a interesting blend of neuroscience, artificial intelligence (AI), cognitive science, and computational modeling. The topics at the focus of this synergy are neuroinformatics, cognitive computing, and computational neuroscience, each providing unique insights and instruments, taken collectively driving our understanding of the human brain and intelligent systems. Neuroinformatics is a framework for brain data management, standardization, and analysis. It provides the computational infrastructure and ontologies necessary to support large-scale, heterogeneous data from neuroimaging, electrophysiology, and genetics (Kennedy, 2016). Global endeavors such as the Human Brain Project (Amunts et al., 2016) and the Neuroscience Information Framework (Gardner et al., 2008) demonstrate how interoperable platforms and open-access data repositories have facilitated reproducible and collaborative research in brain science. At the same time, computational neuroscience employs mathematical and theoretical models as a formulation of neuron and network dynamics, ranging from models of single ion channels to simulations of whole-brain systems (Izhikevich and Edelman, 2008). Computational neuroscience focuses on deriving equations or algorithms when modeling biological mechanisms. This is an important interface between cognition potentially grounded in physiological mechanisms and artificial systems that seek to simulate such processes.

Cognitive computing, which takes inspiration from neurobiological systems, is transforming AI by unifying human-like properties including contextual comprehension, learning, and adaptive reasoning. Cognitive architectures such as IBM's Watson (Ferrucci et al., 2010) and neuromorphic chips like Intel's Loihi (Davies et al., 2018) seek to emulate aspects of human cognitive abilities, combining insights drawn from psychology, neuroscience, and machine learning to create systems that not only calculate but comprehend.

The convergence of these three fields has provided fertile soil for innovations like brain-computer interfaces (BCIs), neurotherapies on an individual basis, and cognitive-robotic hybrid systems. EEG models of stress detection and affective computing (Alarcao and Fonseca, 2017), real-time decoding of decision-making (Cavanagh and Frank, 2014), and neuro-symbolic systems (Besold et al., 2021) highlight the increased explanatory capacity and utility from multi-domain integration.

This collection encompasses of four very varied articles, and a brief about each has been given below:

In the first article (Oyama et al.), the authors developed a predictive-coding inspired variational recurrent neural network (VRNN) that autonomously shifts between focused attention and mind-wandering. The meta-prior parameter w rises when reconstruction error increases, which prompts the network to rely more on internal predictions (mind-wandering), In other case of reduced error, it lowers w, shifting focus back to external sensory input (focused state).The second article (Zeki and Dag) introduce a mathematically reduced discrete-map model for inhibitory neural networks whose bursting behavior is modulated by slow calcium currents. Their model predicts the number of spikes per burst based on initial calcium levels, maps fixed points, and tests stability. It closely matches the behavior of the original continuous system, offering analytical insights into calcium's vital role in shaping neural bursts.

The third article (Li et al.) proposes a novel digital handwriting assessment paradigm for early detection of mild cognitive impairment (MCI) due to Alzheimer's disease (AD). The study was done on 72 subjects (34 healthy controls, 38 MCI due to AD), which collected dynamic handwriting and imagery data via touchscreen and analyzed digital biomarkers from the writing process. Their method achieved AUC = 0.918—substantially outperforming classical MMSE (AUC = 0.783) and MoCA (AUC = 0.859) scales. The technique is intelligent, convenient, and demonstrates strong early-warning potential, though its generalizability across scripts and cultures remains to be verified.

The final article (Luo et al.) highlights the use of a constraint-based metabolic model to investigate bioenergetic disparities between synaptic terminals and neuronal somata in dopaminergic neurons, which are critically implicated in Parkinson's disease (PD). Their model quantifies differential metabolic demands and suggests that synaptic energy metabolism uniquely contributes to neuronal vulnerability in PD. This work connects metabolic modeling with neurodegenerative disease mechanisms and opens avenues for targeted metabolic interventions.

Moving forward, the synergistic collaboration between neuroscientists, computer scientists, data engineers, psychologists, and ethicists will be indispensable. The complexity of cognition demands such pluralism in approach. As we aim to decode the brain and encode intelligence, the integrative spirit of these disciplines must guide our scientific and technological journey. This Research Topic is a call to celebrate and advance this interdisciplinary synergy.

We hope that the reader will find in this Research Topic a useful reference for the state of the art in the emerging field of tools rooted in information theory and applied to neuroscience.
